# Protective Role of Probiotic Supplements in Hepatic Steatosis: A Rat Model Study

**DOI:** 10.1155/2020/5487659

**Published:** 2020-11-06

**Authors:** Aein Azarang, Omid Farshad, Mohammad Mehdi Ommati, Akram Jamshidzadeh, Reza Heydari, Seyedeh Narjes Abootalebi, Ahmad Gholami

**Affiliations:** ^1^Pharmaceutical Sciences Research Center, Shiraz University of Medical Sciences, Shiraz, Iran; ^2^Department of Pharmaceutical Biotechnology, School of Pharmacy, Shiraz University of Medical Sciences, Shiraz, Iran; ^3^Department of Bioinformatics, College of Life Sciences, Shanxi Agricultural University, Taigu, China; ^4^Department of Pharmacology & Toxicology, School of Pharmacy, Shiraz University of Medical Sciences, Shiraz, Iran; ^5^Division of Intensive Care Unit, Department of Pediatrics, School of Medicine, Shiraz University of Medical Sciences, Shiraz, Iran; ^6^Biotechnology Research Center, Shiraz University of Medical Sciences, Shiraz, Iran

## Abstract

**Background:**

Treating nonalcoholic fatty liver disease (NAFLD) is considered one of the public health priorities in the past decade. So far, probiotics have represented promising results in controlling the signs and symptoms of NAFLD. However, attempts to find the ideal probiotic strain are still ongoing. The present study is designed to find the best strain amongst suitable probiotic strains according to their ability to ameliorate histopathological and oxidative stress biomarkers in hepatic steatosis-induced rats.

**Methods:**

Initially, four probiotics species, including *Lactobacillus* (*L.*) *acidophilus*, *L. casei*, *L. reuteri*, and *Bacillus coagulans*, were cultured and prepared as a lyophilized powder for animals. The experiment lasted for fifty days. Initially, hepatic steatosis was induced by excessive ingestion of D-fructose in rats for eight weeks, followed by eight weeks of administering probiotics and D-fructose concurrently. Forty-two six-week-old male rats were alienated to different groups and were supplemented with different probiotics (1∗10^9^ CFU in 500 mL drinking water). After eight weeks, blood and liver samples were taken for further evaluation, and plasma and oxidative stress markers corresponding to liver injuries were examined.

**Results:**

Administration of probiotics over eight weeks reversed hepatic and blood triglyceride concentration and blood glucose levels. Also, probiotics significantly suppressed markers of oxidative stress in the liver tissue.

**Conclusions:**

Although some of the single probiotic formulations were able to mitigate oxidative stress markers, mixtures of probiotics significantly ameliorated more symptoms in the NAFLD animals. This enhanced effect might be due to probiotics' cumulative potential to maintain oxidative stress and deliver improved lipid profiles, liver function markers, and inflammatory markers.

## 1. Background

Nonalcoholic fatty liver disease (NAFLD) is amongst the most prevalent origins of chronic liver disease, which happens to be one of the public health priorities in this century [1]. Despite many efforts and the introduction of several molecular-targeted therapeutic agents, no effective treatments for NAFLD have been provided so far. To provide a new strategy, much attention has been focused on the relationships between NAFLD and the gastrointestinal microbiome [2]. Previous studies have stated that two main risk factors related to NAFLD, e.g., diabetes and obesity, are associated with alterations in the gut microbiome and overgrowth of pathogens in the small intestine [3]. Although the association between the pathology of NAFLD and the gut microbiota is still unknown, it has been found that the microbial overgrowth and their metabolites can lead to overwhelming inflammation due to liver damage [4]. When encountering enteric pathogens, the intestinal epithelium releases inflammatory and proinflammatory cytokines [5]. Also, according to previous studies, an increase in oxidative stress, inflammatory response, and prolipogenic status is observed in NAFLD [6].

Gut microbiota helps the host organisms against pathogens by making a protective barrier and preventing the disruption or loss of intestinal microflora. It is assumed that any alterations in lifestyle and certain dietary habits may trigger some severe disorders such as NAFLD [7, 8]. As stated before, an increase in the intake of energy or refined carbohydrates such as sugar or syrups rich in fructose is observed in most NAFLD cases [9].

The presence of probiotics in appropriate amounts contributes to the general health of the host by creating symbiotic relationships [10]. Probiotics as living and safe organisms which endow many beneficial effects to their hosts and increase their immune system are widely accepted as a natural treatment against metabolic syndromes, diabetes [11], osteoporosis [12], and other related disorders. Literature reviews showed that certain probiotic strains, such as *Lactobacillus* and *Bifidobacterium*, can protect mice against the onset of fructose-induced NAFLD [13]. *Lactobacillus acidophilus* and *Lactobacillus rhamnosus* decrease the levels of faecal TNF-*α* by inhibiting the pathogens binding to the gastrointestinal tract (GI) linings. Also, consistent effects were observed in obese patients with NAFLD who were treated by these two organisms [14]. The effectiveness of probiotics on certain diseases generally depends on many factors, including the bacterial strain, concentration of probiotics, route of administration, age, and diet of the host [15]. As the data about the effectiveness of different types of probiotics are highly diverse and controversial [16], investigating appropriate probiotics for the prevention or treatment of NAFLD can be helpful for both health professionals and the general public.

The consumption of high fructose can cause insulin resistance (IR), excessive production of reactive oxygen species (ROS), hepatic steatosis, liver malfunction, and depletion of the hepatocyte population [17, 18]. Oxidative stress, induced by a high-energy diet, builds up to the genesis and progression of steatohepatitis from steatosis [19]. Based on previous studies, oxidative stress is implicated in the pathogenesis of NAFLD [20]. There have been several animal and clinical studies that clarify correlations between conducted tests, including oxidative stress biomarkers and liver pathogenicity [21].

This research is aimed at examining the impacts of some probiotic species on fatty acid profile and liver functions in the hepatic steatosis rats in order to understand the protective role of probiotics in the prevention and genesis of liver dysfunctions, especially in NAFLD. We were mainly focused on the effects of single and mixture formulations of these probiotics in NAFLD. Different results regarding the administration of probiotics and oxidative stress markers are highlighted and discussed. This research may help answer questions regarding the role of the abovementioned probiotics in the prevention of NAFLD in rats.

## 2. Methods

### 2.1. Chemicals

A list of the chemicals used is provided in the supplementary table (Table S1). Kits used for the examination of the hepatic malfunction biomarkers such as triglyceride (TG), alkaline phosphatase (ALT), and glucose were purchased from Pars Azmun® Co. (Iran). Materials used for the buffer preparations were purchased from Merck KGaA (Darmstadt, Germany). All cultures used for bacterial inoculum preparation, including De Man, Rogosa, and Sharpe (MRS) agar, L-S differential (LS), trypticase soy powders were obtained from Sigma-Aldrich (St. Louis, MO, USA).

### 2.2. Microbial Identification

Initially, 20 different samples of traditional fermented yoghurt were gathered from the north coast of the Persian Gulf. The samples were kept at 4°C. Ten grams of each yoghurt sample was homogenized using a laboratory mixer after being diluted in peptone solution (4%) and sterilized water. LS medium, as a differential medium for the growth of *Streptococcus*, and *Lactobacillus* were used. For the specific isolation of lactobacilli, MRS agar was employed.

After the incubation of plates under anaerobic conditions for three days (at 37°C), the isolates were identified according to their biochemical, cultural, and morphological properties based on Bergey's Manual of Systematic Bacteriology [22]. Several biochemical tests, including the Voges-Proskauer (VP) test, nitrate reduction, resistance to bile salts, sugar fermentation, and motility were conducted on the probiotics in order to confirm the bacterial strains (Table 1) [23].

Also, 16S rRNA gene sequence analysis was done for the molecular identification of isolates. The purified isolates were diluted in a saline solution. Then, the isolates were centrifugated at 4500 g for 10 min using a refrigerated laboratory centrifuge and washed several times before preparing for PCR amplification. Heat shock method was used for DNA extraction, and two universal forward and reverse primers were used for amplification of 16SrDNA sequence of bacteria with the following sequences:

- F: 5′-ACGGGCGGTGTGTAC-3′
- R: 5′-CAGCCGCGGTAATAC-3′

Amplification buffer contains 4 ng whole genome of bacteria, 2.5 units of DNA polymerase (Taq), 400 nM of each primer, and excess dNTP which finally reaches to 50 *μ*L. Routine PCR protocol, according to Gholami et al. [24], was applied for the amplification process during 20 cycles. The PCR products were then placed in a Tris/borate/ethylene-diamine tetra-acetic acid (EDTA) buffer containing 1 *μ*g/mL ethidium bromide and electrophoresed using 1% agarose gel and visualized by a UV apparatus. The DNA sequence, which was about 800 bp in length, was retrieved by a DNA gel purification kit (AccuPrep®, Bioneer, Korea) and sent to the CinnaGen Co. for sequencing determination [24]. The obtained sequence was analyzed using bioinformatic tools in the NCBI database and finally submitted after performing the steps. Accordingly, the isolated strains were identified, and four probiotic strains, including *Lactobacillus acidophilus*, *Lactobacillus casei*, *Lactobacillus reuteri*, and *Bacillus coagulans* were selected for animal studies.

### 2.3. Formulations

Four different probiotic strains, namely, *Lactobacillus acidophilus*, *Lactobacillus casei*, *Lactobacillus reuteri*, *Bacillus coagulans*, and a mixture of these strains were cultured, centrifugated, freeze-dried, and designed at a concentration of 1 × 10^9^ probiotics/mL. The probiotic strains were freshly prepared every day and dispersed in drinking water containing 20% of D-fructose.

### 2.4. Experimental Animals

Forty-two, male, 42-day-old Sprague-Dawley rats (av. weight of about 90 g) were obtained from the Razi Institute in Shiraz, Iran. Rats were observed for several days after their arrival to the laboratory and given seven days to familiarize themselves with their new setting. They were placed alone in metal cages at controlled room temperature, and their living conditions were considered as a 12-hour light-dark cycle with a humidity of around 50%. The animals were then randomly divided into the following seven experimental groups:

- Group 1: received *Lactobacillus acidophilus* + high-fructose regimen
- Group 2: received *Bacillus coagulans* + high-fructose regimen
- Group 3: received *Lactobacillus casei* + high-fructose regimen
- Group 4: received *Lactobacillus reuteri* + high-fructose regimen
- Group 5: received a mixture of the mentioned probiotics inclusive of *Lactobacillus acidophilus*, *Lactobacillus casei*, *Lactobacillus reuteri*, and *Bacillus coagulans* + high-fructose regimen
- Group 6: received high-fructose regimen
- Group 7: received drinking water

Group 6 was considered a positive control and group 7 a negative control.

All rats were exposed to standard diet and water concurrently with no limitations. Food and water did not differ between different groups and were monitored. This study was conducted at the Center of Comprehensive Experimental Medicine, Shiraz, Iran, from November 2018 until March 2019.

### 2.5. Diet Preparations

Initially, daily water consumption was measured, and the average was calculated. Each rat drank about 70 mL of water daily. In the beginning, 15 mg D-fructose was allocated to each rat and diluted in the drinking water once a day, freshly made every day. As the rats got more massive, the amount was calculated based on the weight/volume formula. Each rat received a minimum of 1∗10^9^ CFU/mL of probiotics (single or mixed) in drinking water containing 20% of D-fructose freshly prepared every day before noon. D − fructose > 99% (Merck KGaA, Darmstadt, Germany) was used in drinking water to induce NAFLD. Drinking water containing fructose was freshly prepared and administered every day based on the weight/volume formula.

Rats had free access to a standard pellet diet consisting of 20% protein; 6.0% fat; 10.0% crude fibre; 5.0% crude ash; 0.6% calcium; 0.4% phosphorus; 0.9% sodium; and 0.5-1% moisture and other nutritional additives inclusive of vitamin A, vitamin D3, manganese, zinc, and selenium. A 15-gram portion of this pellet has 60 calories.

### 2.6. Surgical Protocol

This experiment lasted for sixteen weeks, and on the last day, rats were euthanized after being anaesthetized by injection of 80 mg/kg thiopental intraperitoneally. It was ensured that unconsciousness persisted until death occurred with minimum pain and distress. All animals were behaved and sacrificed following the National Institutes of Health guide for the care and use of laboratory animals (NIH Publications No. 8023, revised 1978). The experimental procedures were all performed according to the approval of the Ethical Committee of Shiraz University of Medical Sciences under code no. 97-01-33-15624.

### 2.7. Blood and Tissue Sampling

The liver and blood samples were gathered from rats anaesthetized by intraperitoneal injection of thiopental at the end of the experiment. The blood samples, obtained from the inferior vena cava, were put in the gel separator/clot activator vacuum tubes. To obtain blood serum, all blood samples were centrifuged (15 min at 4°C, 5000 g). A standard diagnostic kit (Pars Azmun® Co., Iran) and an autoanalyzer (Mindray BS-200®, China) were applied for measuring ALT, TG, and glucose levels [25]. During the experiment, each animal's body weight was monitored every week, and any weight change was recorded.

### 2.8. Oxidative Stress Assays

#### 2.8.1. The Antioxidant Power of the Liver

Ferric-reducing/antioxidant power (FRAP) assay was applied to calculate the antioxidant capacity of the animal liver after the consumption of probiotics [26]. FRAP reagent is composed of three solutions, including the TPTZ solution, the 20 mmol/L ferric chloride solution, and the 300 mmol/L acetate buffer (pH = 3.6), that are combined at a ratio of 1/1/10, respectively. The TPTZ solution was prepared by mixing 10 mmol/L TPTZ in 40 mmol/L hydrochloric acid. All solutions were freshly made just before the assay was conducted.

A total of 500 mg liver tissues was dissolved in Tris-HCl buffer. After homogenization, 50 *μ*L of homogenates was dissolved in 150 *μ*L deionized water and transferred to 1.5 mL of the FRAP solution and incubated in a dark environment (37°C for 5 min). In this study, absorbance was measured using a microplate ELIZA reader (BioTek®, US) at 593 nm.

#### 2.8.2. Liver Glutathione Assay

To measure the liver glutathione content, initially, 200 mg of liver tissue samples was added to the EDTA solution (8 mL, 40 mmol/L) and conducted to the homogenization process [27]. A total of 5 mL samples was added to 4 mL of distilled water and TCA (1 mL, 50% *w*/*v*) at 4°C. After vortexing and centrifugating at 10000 g for 15 min using a refrigerated centrifuge, this was followed by mixing the supernatant with a solution consisting of Tris-HCl buffer (4 mL), DTNB (100 *μ*L), and 1 mL methanol. A microplate ELIZA reader (BioTek®, USA) was used to measure the absorbance of the solution at 412 nm.

#### 2.8.3. Peroxidation of Liver Lipids

A total of 500 mg homogenate liver samples was dissolved in potassium chloride solution at 4°C and transported to a solution containing thiobarbiturate and phosphoric (V) acid at a ratio of 1 : 3 *v*/*v*. After boiling the mixture for 45 minutes, vigorous mixing was applied to add 2 mL n-butanol, and the liver specimens were centrifugated at 10000 g for 5 min. A microplate ELIZA reader (BioTek®, USA) was used to measure the absorbance of the sample at 532 nm [28].

#### 2.8.4. Liver ROS

According to Jamshidzadeh et al., 200 mg of liver specimens was dissolved in Tris-HCl buffer at a ratio of 1 : 10 *w*/*v* at 4°C; then, this was added to 5 *μ*L DCFH-DA (10 mmol/L) and incubated for 30 minutes at 37°C. A FLUOstar Omega® Microplate Reader (Germany) was used to measure the intensity of fluorescence of the samples at *λ*_excitation_ = 485 nm and *λ*_emission_ = 525 nm [29].

### 2.9. Carbonylation of Liver Proteins

To measure the carbonylated protein level of the hepatic tissues, according to Colombo et al., a spectrophotometric test was applied when the liver proteins were carbonylated by the assistance of 2,4-dinitrophenyl hydrazine (DNPH) [30]. A total of 0.5 g liver specimens was homogenized in 0.1 M sodium phosphate buffer (containing 0.1% Triton X-100, pH = 7.4) and a sample of 0.5 mL of the liver homogenate was added to 0.5 mL of 0.1% DNPH (*w*/*v* in 2.0 N HCl). This mixture was incubated for 60 minutes in the dark at 24°C. Total liver proteins were precipitated by the addition of 0.5 mL TCA, and then, this mixture was quickly centrifugated at 10000 g for 5 min. The biomass was collected, and the supernatant was washed several times and discarded using a 1 mL ethanol : ethyl acetate solution; then, the pellets were dispersed again in 1 mL Tris buffer. An Ultrospec 2000® Spectrophotometer (Pharmacia Biotech, Sweden) was used to record the absorbance at 370 nm.

### 2.10. Statistical Analysis

The data were statistically analyzed using a one-way analysis of variance (one-way ANOVA), followed by the Tukey method for post hoc analysis, using SPSS IBM software version 23, and the results were displayed as mean ± SEM. Values were considered significant when *P* < 0.05.

## 3. Results

### 3.1. Biochemical and Molecular Tests for Bacterial Identification

After conducting several identification tests, the results indicated that the nitrate reduction test was negative for all the strains, and the test for determining the resistance to bile salts showed positive results for all. The VP test was positive for *Lactobacillus reuteri* and *Bacillus coagulans* strains and negative for all the other strains. The *Bacillus coagulans* strain showed positive results for the motility test (Table 1). Also, the PCR sequences were examined and then submitted in the NCBI databases under accession numbers MN658702, MN658703, MN658704, and MN658705. The similarity of the sequences was analyzed using the BLAST bioinformatics tools. According to all identification tests, four microbial probiotic isolates, including *L. acidophilus*, *L. casei*, *L. reuteri*, and *B. coagulans*, were found. Other strain designation assays including physiological and molecular analyses as well as probiotics and safety property assays such as acid and bile tolerance, antibacterial activity, antibiotic susceptibility testing, catalase, and hemolytic test and MTT assay were previously tested and reported by our team [12, 23].

### 3.2. Animal Studies

**Figure 1:**
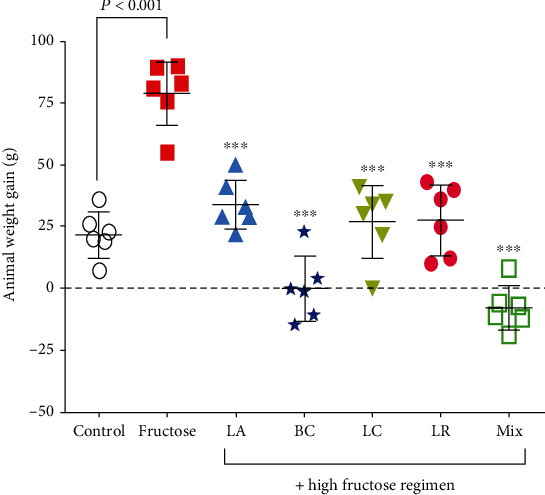
Animal weight gain in NAFLD rats and the effect of probiotic administration. Data are demonstrated as mean ± SEM (*n* = 6). Ctrl: control; LA: *Lactobacillus acidophilus*; BC: *Bacillus coagulans*; LC: *Lactobacillus casei*; LR: *Lactobacillus reuteri*; Mix: a mixture of probiotics including *Lactobacillus acidophilus*, *Lactobacillus casei*, *Lactobacillus reuteri*, and *Bacillus coagulans*. ∗∗∗ indicates a significant difference from the fructose control group (*P* < 0.001).

According to Figure 1, the weight of the animals increased by 77.2 ± 6% in the high-fructose diet (HFD) group (positive group) which had been significantly increased compared to the negative control group (*P* < 0.05). Mean body mass in groups 1, 2, 3, and 4 with a high-fructose plus probiotic diet were lower with 62.5 ± 6%, 80 ± 7%, 68.8 ± 5%, and 69 ± 6% differences, respectively, which had been significantly decreased compared to the HFD-only group (*P* < 0.05). Also, group 5, which received a mixture of probiotics, had a much lower mean body mass with a difference of 85% compared to the HFD-only group (*P* < 0.05).

**Figure 2:**
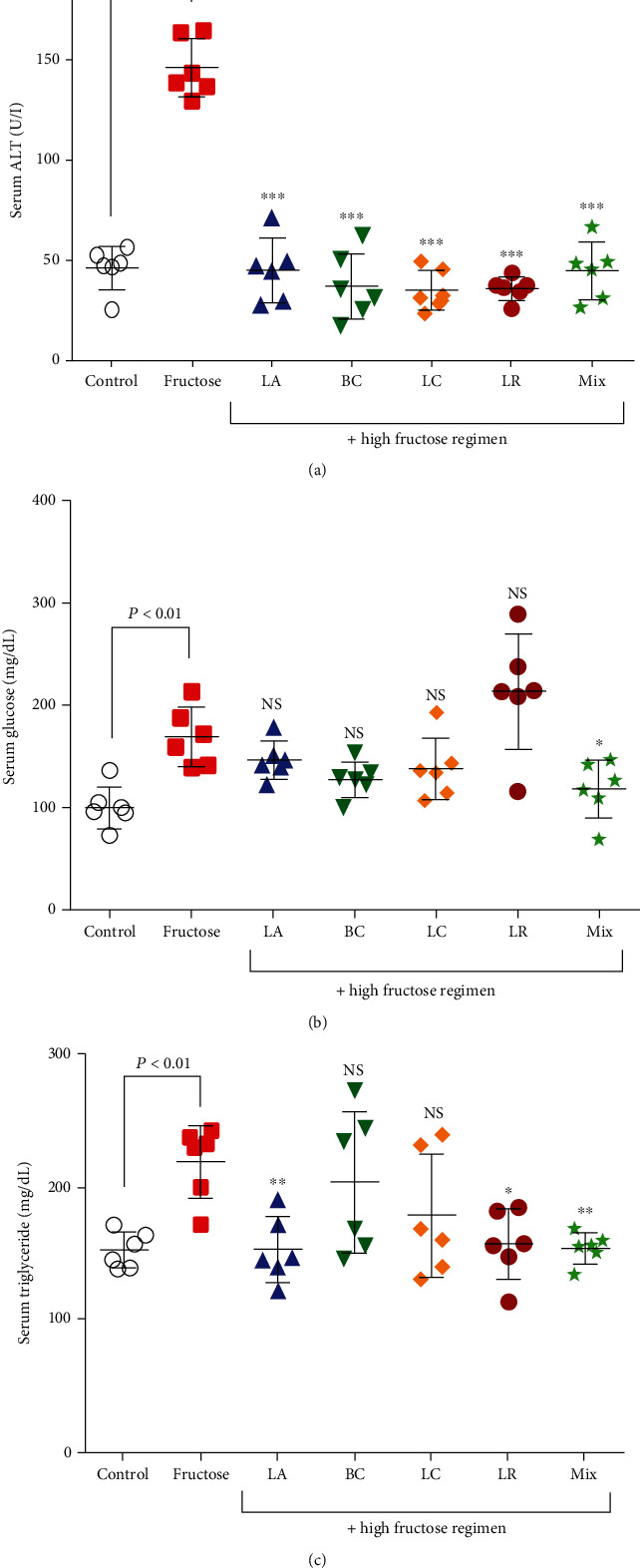
Serum biochemical changes in NAFLD rats and the effects of probiotic administration. (a) Serum alanine aminotransferase (ALT) test, (b) serum triglyceride level, and (c) serum glucose level. Data are demonstrated as mean ± SEM (*n* = 6). Ctrl: control; LA: *Lactobacillus acidophilus*; BC: *Bacillus coagulans*; LC: *Lactobacillus casei*; LR: *Lactobacillus reuteri*; Mix: a mixture of probiotics including *Lactobacillus acidophilus*, *Lactobacillus casei*, *Lactobacillus reuteri*, and *Bacillus coagulans*; ns: not significant. ∗ indicates a significant difference from the fructose control group (*P* < 0.05). ∗∗ indicates a significant difference from the fructose control group (*P* < 0.01). ∗∗∗ indicates a significant difference from the fructose control group (*P* < 0.001).

As indicated in Figure 2(a), serum ALT levels had a 67.5 ± 5% increase in the high-fructose regimen group compared to the negative control group (*P* < 0.05). Serum ALT levels in groups 1, 2, 3, 4, and 5 receiving probiotics plus HFD-diet were significantly lower with a difference of 67.5 ± 5%, 69.5 ± 4%, 70.2 ± 5%, 69.5 ± 3%, and 67.5 ± 2%, respectively, compared to the HFD-only rats (*P* < 0.05).

As has been demonstrated in Figure 2(b), the serum triglyceride levels in the HFD-only group had a 31.8 ± 4% increase compared to the negative control group (*P* < 0.05). Serum triglyceride levels were significantly lower in the HFD plus probiotic groups compared to the HFD-only group and had differences of 32 ± 4%, 32.8 ± 5%, and 33 ± 2% in groups 1, 4, and 5, respectively (*P* < 0.05). The changes in groups 2 and 3 were nonsignificant (*P* < 0.05).

The serum glucose level in the HFD-only group was significantly increased by 80 ± 4% in comparison to the negative control group and had nonsignificant results in groups 1, 2, 3, and 4 (*P* < 0.05). However, the serum glucose level in the fifth group, which received a mixture of probiotics plus an HFD, was significantly lower with a difference of 33.3 ± 4% compared to the HFD-only group (Figure 2(c), *P* < 0.05).

**Figure 3:**
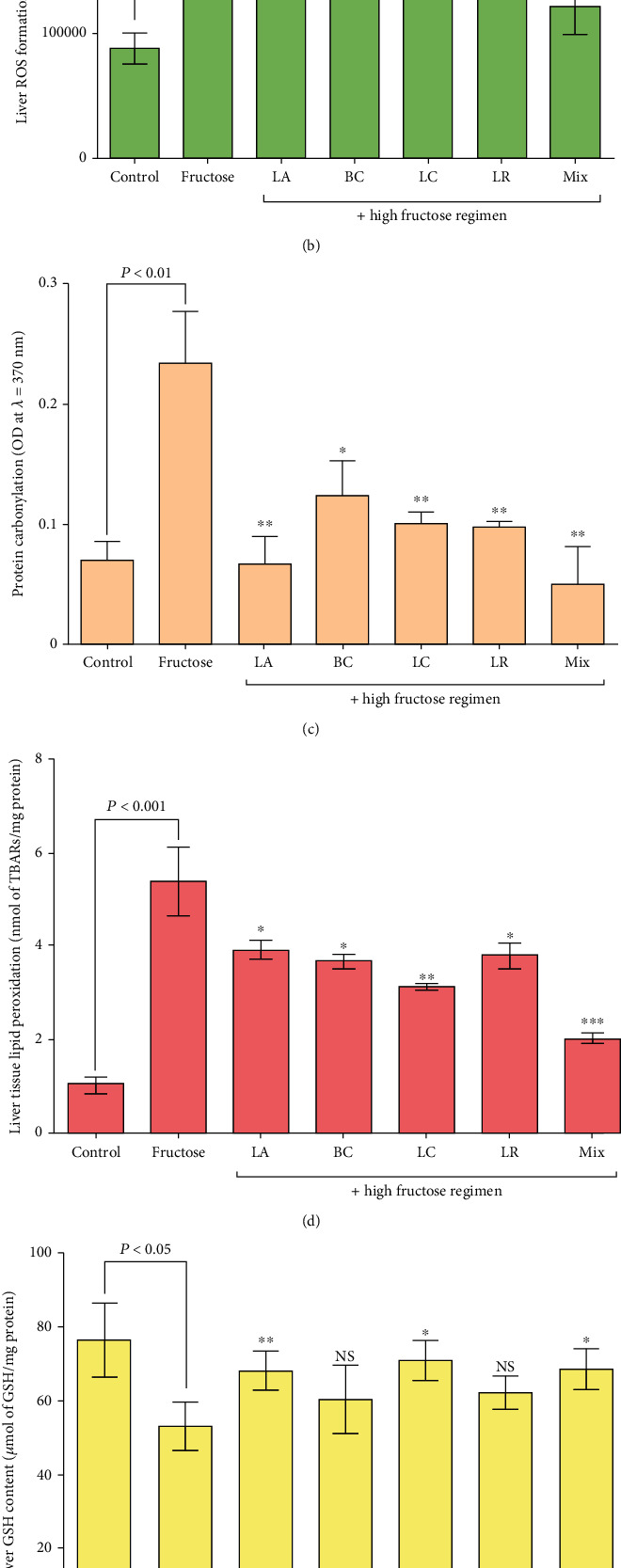
Oxidative stress markers in the liver tissue of NAFLD rats and effects of probiotic administration. Data are demonstrated as mean ± SEM (*n* = 6). (a) Liver tissue ferric-reducing/antioxidant power (FRAP) assay, (b) liver reactive oxygen species (ROS) formation, (c) liver protein carbonylation, (d) liver tissue lipid peroxidation, (e) hepatic glutathione content, and (f) liver tissue triglyceride level. Ctrl: control; LA: *Lactobacillus acidophilus*; BC: *Bacillus coagulans*; LC: *Lactobacillus casei*; LR: *Lactobacillus reuteri*; Mix: a mixture of probiotics inclusive of *Lactobacillus acidophilus*, *Lactobacillus casei*, *Lactobacillus reuteri*, and *Bacillus coagulans*; ns: not significant. ∗ indicates a significant difference from the fructose control group (*P* < 0.05). ∗∗ indicates a significant difference from the fructose control group (*P* < 0.01). ∗∗∗ indicates a significant difference from the fructose control group (*P* < 0.001).

The total antioxidant level in the liver tissue of group 5 showed a 36 ± 2% increase compared to the HFD-only group which had been statistically significant; also, the total antioxidant level in the HFD-only group was significantly lowered by 45 ± 4% in comparison to the negative control group (*P* < 0.05). No significant modifications occurred in all the other testing groups (Figure 3(a), *P* < 0.05).

According to Figure 3(b), the formation of reactive oxygen species (ROS) was significantly lowered with a difference of 20.8 ± 2%, 33.3 ± 3%, 37.5 ± 2%, and 50 ± 3% in groups 1, 2, 3, and 5, respectively, compared to the HFD-only group (*P* < 0.05). No significant modifications were noted in group 4, and an increase of 82.5 ± 2% in ROS formation occurred in the HFD-only group in comparison to the negative control group (*P* < 0.05).

The protein-carbonylation level of the liver tissue in groups that received probiotics plus an HFD was significantly decreased by 67.1 ± 3%, 47.8 ± 3%, 56.5 ± 1%, 57.3 ± 0.5%, and 78.2 ± 3% in groups 1, 2, 3, 4, and 5, respectively, in comparison to the HFD-only group (*P* < 0.05). This item was increased by 67.3 ± 5% in the HFD-only group compared to the control group (Figure 3(c), *P* < 0.05).

The lipid peroxidation levels were significantly lowered by 31 ± 1.5%, 32.8 ± 1%, 46.6 ± 0.5%, 31 ± 1%, and 62 ± 1%, respectively, in groups 1, 2, 3, 4, and 5, which received an HFD plus probiotic strains, compared to the HFD-only group (*P* < 0.05). This item was significantly increased by 82.8 ± 4% in the HFD-only group in comparison to the negative control group (Figure 3(d), *P* < 0.05).

The liver glutathione content in the HFD-only group was significantly decreased by 29.5 ± 3% compared to the negative control group and increased by 21.4 ± 2%, 25.6 ± 2%, and 23.6 ± 2% in groups 1, 3, and 5, respectively, compared to the HFD-only group (*P* < 0.05). The glutathione content did not have any significant changes in groups 2 and 4 (Figure 3(e), *P* < 0.05).

The liver tissue triglyceride levels were lowered with differences of 52 ± 2%, 61.6 ± 3%, and 60 ± 2% in groups 1, 4, and 5, compared to the HFD-only group, respectively, which was statistically significant (Figure 3(f), *P* < 0.05). Liver tissue triglyceride levels increased by 92 ± 6% in the HFD-only group compared to the negative control group (*P* < 0.05). The changes in groups 2 and 3 were nonsignificant (*P* < 0.05).

## 4. Discussion

In the past decade, research on probiotics has attracted much attention due to their protective role in NAFLD. The results of a research conducted by Yadav et al. showed that the probiotic “Dahi” containing *Lactobacillus acidophilus* and *Lactobacillus casei* improved parameters such as blood glucose and triglyceride levels and decreased the high-density lipoprotein cholesterol in animals suffering from metabolic syndrome [31]. Probiotics used in our study were gathered from organic yoghurts made in unique, organic dishes made from clay. Yoghurts were gathered from areas around the Persian Gulf and villages of Fars province, which have always been famous for having beneficial health effects, and people have been using them for years for different health complications such as diarrhoea, fatty liver diseases, or weight loss [32]. A majority of the locals believe that this type of yoghurt can prevent illnesses, but no experiments had supported this idea up until now [33]. On that account, initially, the isolated strains were characterized and confirmed for possessing probiotic features [12, 23, 34]. In these studies, the properties of isolated and selected probiotics were rigorously examined. These tests were inclusive of morphological, physiological, and biochemical properties as well as 16SrDNA, acid and bile tolerance, antimicrobial activity, hemolytic activity, protease activity, cell surface hydrophobicity, and autoaggregation [23]. When samples were gathered, probiotic strains were isolated and used solely or as a mixture in this study. It is worth adding that these probiotics were tested before use, and none of them showed any characteristics of being pathogenic or harmful to humans/mammals. Animal and human studies showed that the composition of gut microbiota was significantly changed in NAFLD, and the beneficial effects of probiotic supplements in compensating were frequently approved [35–38].

Several systematic reviews and meta-analysis have mentioned that the potential of probiotics is disease- and strain-specific [16, 39, 40]. Up to now, most studies have focused on *Lactobacillus* and *Bifidobacterium* strains. In this study, the effects of *Lactobacillus acidophilus*, *Lactobacillus casei*, *Lactobacillus reuteri*, and *Bacillus coagulans* were compared together and with the mixture of the mentioned probiotics in NAFLD-induced male Sprague-Dawley rats.

This study examined some of the critical serum and histopathological markers related to NAFLD, including mean body weight, serum ALT, serum TG, serum glucose, liver TG content, liver glutathione content and FRAP, ROS, protein carbonylation, and lipid peroxidation of the liver tissue. According to our results, probiotics were able to change the mentioned parameters during the experiment. Upon the administration of probiotics, improved disease markers such as serum TG, serum glucose, and ALT levels and improved oxidative stress markers were observed. Conservation can probably explain improved liver triglyceride levels in the expression and activity of the transcription factor peroxisome proliferator-activated receptor-*α* (PPAR-alpha). There are some studies indicating that substances binding to PPAR-alpha induced its activation to bind to DNA, and stimulated the expression of proteins involved in the metabolism of fatty acids [41, 42].

Also, oxidative stress has been reportedly associated with the pathogenesis of NAFLD [43, 44], and the improved results reveal the critical role of probiotics in the prevention of NAFLD. Based on previous studies, oxidative stress may lead to the genesis of steatohepatitis from steatosis caused by a high-calorie diet [19]. Therefore, amelioration of oxidative stress markers along with the reduction of free radicals and inflammation in the liver tissue could be the factors that contribute to liver pathology based on previous experiments [45, 46]. As stated in the previous studies, the changes observed in the antioxidant response, the hepatoprotective effects, could be due to an increase in the expression and activity of the transcription factor Nrf2 [47].

Based on previous studies [48], animals fed with a high-fructose diet had elevated serum lipid profiles such as TG, serum ALT, and serum glucose compared to animals with a standard diet. The increased levels of lipids may be linked to the accumulation of fat droplets in animals receiving a high-fructose diet, leading to the genesis of hepatic steatosis and different stages of NAFLD [49]. High-fructose diets can alter the gut microbiota, leading to gut permeability, decreased bacterial LPS removal, and increased metabolic endotoxemia [50]. According to previous studies [51], endotoxemia can lead to various metabolic dysfunctions resulting in disturbance of fat metabolism. This incidence may eventually cause an increase in fatty acid uptake, fat disposition in the hepatic tissues, and hepatic steatosis. Also, a *Bacillus* species called *Bacillus coagulans*, primarily considered as a probiotic strain, had interestingly attenuated the development of NAFLD. This strain significantly prevented the weight gain in NAFLD-induced rats and substantially lowered free radicals that may contribute to inflammatory and metabolic diseases. *Bacillus coagulans* is a sporogenic bacteria which is essential from an industrial point of view because of its resistance to strong gastric acid and high temperatures. Recent studies on the probiotic effects of *Bacillus* species have been focused on the prevention and treatment of metabolic disorders [52]. Some probiotic-containing supplements controlled weight gain and hyperglycemia induced in animals by a high-fructose diet [53]. Moreover, a combination of soya pulp and *Bacillus coagulans* demonstrated improved bile acid levels in metabolic dysfunctions and NAFLD diseases [54].

This study primarily focuses on answering the critical question of which probiotic strains to use in the prevention of NAFLD. Based on recent studies, probiotics have been shown to have diverse effects on different organs and hosts and proven to have the potential to be disease- and strain-specific, as mentioned in the manuscript [16, 55]. It is believed that isolated strains of probiotics from different origins may have different results. Although each strain used in this study was able to overcome some implications of nonalcoholic fatty liver disease, our results indicated that a mixture of these probiotic strains had an overall better outcome in all the tested markers and was considered as the best-treating group. Using the mixture of probiotic strains seemed to help to maintain or to preserve the lipid profiles and to reduce the chance of hepatic steatosis in group 6, which received a high-fructose regimen. It has been proved that using a mixture of probiotic strains is highly beneficial for the host's health rather than a single-strain probiotic in many cases [56]. Animals with NAFLD displayed improvement in several disease markers and amelioration of the metabolic syndrome after receiving a mixture of probiotic strains [57]. This might be due to their presumed complementary and synergistic effects, especially within the gut [58].

Moreover, the observed hepatoprotective effects may be influencing mitochondrial functions, which is one of the most relevant causes in the prevention of hepatic steatosis [59]. We observed the same results regarding the consumption of a mixture of probiotics. In this case, we had used four different species, inclusive of *Lactobacillus acidophilus*, *Lactobacillus casei*, *Lactobacillus reuteri*, and *Bacillus coagulans* that had not been used together in any other studies.

Therefore, these results showed that a cocktail mixture of probiotics could be considered a promising combination for the prevention of NAFLD. Although rat models with a nutritional deficiency to develop NAFLD are usually preferred due to their natural preference and vital importance in illuminating the pathophysiological mechanisms of NAFLD, it is essential to note that translation of animal-study results to the human population has failed frequently [60]. The results of this study have provided significant evidence for the role of probiotics in the prevention of NAFLD; nevertheless, more randomized clinical trials are needed to confirm their role in humans. On the whole, due to probiotics' relatively low production cost, low side effects, availability, and limitations that are not difficult to overcome, further clinical studies can illuminate the effects of probiotics in the human body.

## 5. Conclusion

Since different probiotic strains exert diverse effects on metabolic disorders, future studies should include an emphasis on the interactions between probiotics and NAFLD. This experiment used four different probiotic strains, and the results showed that the mixture of the mentioned probiotic strains was more effective in the prophylaxis of NAFLD than single-strain therapy. Improved lipid profiles, liver function markers, and inflammatory marker levels supported our theory. Having nutritional and therapeutic potentials, probiotics were able to control and prevent hepatic steatosis and similar disorders.

We believe a more detailed investigation regarding specific microbiome profiles of the GI in NAFLD patients may allow better-individualized modulation of the disease by probiotics. Further studies must be focused on the potentially suitable probiotic mixtures, concentrations, intervals, and algorithms of administration in human clinical trials.

## Figures and Tables

**Table 1:** The biochemical and physiological characteristics of the probiotics used in this study.

*Test*	*B. coagulans*	*L. reuteri*	*L. acidophilus*	*L. casei*
Motility	*Yes*	*No*	*No*	*No*
Catalyze	*Yes*	*No*	*No*	*No*
Oxidase	*No*	*Yes*	*No*	*Yes*
Lactose	*No*	*Yes*	*No*	*Yes*
Fructose	*Yes*	*No*	*Yes*	*Yes*
Glucose	*Yes*	*Yes*	*Yes*	*Yes*
Galactose	*Yes*	*No*	*No*	*Yes*
Cellobiose	*Yes*	*No*	*Yes*	*Yes*
Sorbose	*No*	*No*	*No*	*No*
Maltose	*Yes*	*Yes*	*Yes*	*Yes*
Sucrose	*Yes*	*Yes*	*Yes*	*Yes*
Mannose	*Yes*	*No*	*Yes*	*Yes*
Cellulose	*No*	*Yes*	*No*	*No*
Trehalose	*No*	*No*	*Yes*	*Yes*
Xylose	*No*	*Yes*	*No*	*No*
Melezitose	*Yes*	*No*	*Yes*	*Yes*
Melibiose	*No*	*Yes*	*No*	*Yes*
Arabinose	*Yes*	*Yes*	*Yes*	*No*
Ribose	*Yes*	*Yes*	*Yes*	*Yes*
Raffinose	*No*	*No*	*Yes*	*No*
VP	*Yes*	*Yes*	*No*	*No*
Nitrate reduction	*No*	*No*	*No*	*No*
Gas production from glucose	*No*	*Yes*	*No*	*Yes*
Resistance to bile salts	*Yes*	*Yes*	*Yes*	*Yes*
Growth at 15°C	*Yes*	*Yes*	*No*	*Yes*
Growth at 45 °C	*Yes*	*Yes*	*No*	*Yes*
Motility	*Yes*	*No*	*No*	*No*
Catalyze	*Yes*	*No*	*No*	*No*
Oxidase	*No*	*Yes*	*No*	*Yes*
Lactose	*No*	*Yes*	*No*	*Yes*
Fructose	*Yes*	*No*	*Yes*	*Yes*
Glucose	*Yes*	*Yes*	*Yes*	*Yes*
Galactose	*Yes*	*No*	*No*	*Yes*
Cellobiose	*Yes*	*No*	*Yes*	*Yes*
Sorbose	*No*	*No*	*No*	*No*
Maltose	*Yes*	*Yes*	*Yes*	*Yes*
Sucrose	*Yes*	*Yes*	*Yes*	*Yes*
Mannose	*Yes*	*No*	*Yes*	*Yes*
Cellulose	*No*	*Yes*	*No*	*No*
Trehalose	*No*	*No*	*Yes*	*Yes*
Xylose	*No*	*Yes*	*No*	*No*
Melezitose	*Yes*	*No*	*Yes*	*Yes*
Melibiose	*No*	*Yes*	*No*	*Yes*
Arabinose	*Yes*	*Yes*	*Yes*	*No*
Ribose	*Yes*	*Yes*	*Yes*	*Yes*
Raffinose	*No*	*No*	*Yes*	*No*
VP	*Yes*	*Yes*	*No*	*No*
Nitrate reduction	*No*	*No*	*No*	*No*
Gas production from glucose	*No*	*Yes*	*No*	*Yes*
Resistance to bile salts	*Yes*	*Yes*	*Yes*	*Yes*
Growth at 15 °C	*Yes*	*Yes*	*No*	*Yes*
Growth at 45 °C	*Yes*	*Yes*	*No*	*Yes*

## Data Availability

The datasets used and/or analyzed during the current study are available from the corresponding author on reasonable request.

## Abbreviations

NAFLD: Nonalcoholic fatty liver disease
GI: Gastrointestinal tract
TG: Triglyceride
ALT: Alkaline phosphatase
TCA: Trichloroacetic acid
DTNB: 5,5-Dithionitro-benzoic acid
DNPH: 2,4-Dinitrophenylhydrazine
DCF-DA: 2,7-Dichlorofluorescein
HCl: Hydrochloric acid
EDTA: Ethylene-diamine tetra-acetic acid
TPTZ: 2,4,6-Tri(2-pyridyl)-s-triazine
Tris-HCl: 2-Amino-2-hydroxymethyl-propane-1,3-diol-hydrochloride
MRS: De Man, Rogosa, and Sharpe
LS: L-S differential medium
VP: Voges-Proskauer
FRAP: Liver ferric-reducing antioxidant power
MTT: 3-(4,5-Dimethylthiazol-2-yl)-2,5-diphenyltetrazolium bromide
ROS: Reactive oxygen species.

## Supplementary Materials

Supplementary Materials Supplementary Table 1: list of the chemicals and manufacturing companies
