# Supplementary material for: Protective Role of Probiotic Supplements in Hepatic Steatosis: A Rat Model Study
Source: Biomed Res Int. 2020 Nov 6;2020:5487659. doi: 10.1155/2020/5487659 (PMC7704153; doi:10.1155/2020/5487659)
Supplement: Supplementary Materials — Supplementary Table 1: list of the chemicals and manufacturing companies [file 5487659.f1.docx]

| Chemicals | Manufacturer company | country |
| --- | --- | --- |
| n-Banutol | Merck | Germany |
| guanidine-HCl, | Merck | Germany |
| Citric acid | Merck | Germany |
| guanidine-HCl | Merck | Germany |
| thiobarbituric acid (TBA) | Merck | Germany |
| 5,5-Dithionitro-benzoic acid (DTNB) | Merck | Germany |
| 2,4-dinitrophenylhydrazine (DNPH) | Merck | Germany |
| Potassium chloride (KCL) | Merck | Germany |
| *Na_2_HPO_4_* | Merck | Germany |
| meta-Phosphoric acid | Merck | Germany |
| ethanol | Merck | Germany |
| Ethyl acetate, 2,7- dichloro fluorescein diacetate (DCF-DA) | Merck | Germany |
| dichlorofluorescein diacetate (DCF-D) | Merck | Germany |
| Ethylene-diamine tetra-acetic acid *(EDTA)* | Merck | Germany |
| Trichloroacetic acid (TCA) | Merck | Germany |
| Ferric chloride | Merck | Germany |
| 2,4,6-Tri(2-pyridyl)-s-triazine (TPTZ) | Merck | Germany |
| thiopenthal | Merck | Germany |
| Sodium citrate | Merck | Germany |
| D-Fructose | Merck | Germany |
| Triton X-100 | Merck | Germany |
| p-Dimethylamino benzaldehyde | Merck | Germany |
| n-Propanol | Merck | Germany |
| 2-amino-2-hydroxymethyl-propane-1,3-diol-Hydrochlorid *(Tris-HCl* | Merck | Germany |
